# Collagen type XV and the ‘osteogenic status’

**DOI:** 10.1111/jcmm.13137

**Published:** 2017-03-22

**Authors:** Gina Lisignoli, Elisabetta Lambertini, Cristina Manferdini, Elena Gabusi, Letizia Penolazzi, Francesca Paolella, Marco Angelozzi, Veronica Casagranda, Roberta Piva

**Affiliations:** ^1^ Laboratorio di Immunoreumatologia e Rigenerazione Tissutale Istituto Ortopedico Rizzoli Bologna Italy; ^2^ Department of Biomedical and Specialty Surgical Sciences University of Ferrara Ferrara Italy

**Keywords:** collagen XV, osteogenesis, mesenchymal stromal cells, osteoblasts, mineralization

## Abstract

We have previously demonstrated that collagen type XV (ColXV) is a novel bone extracellular matrix (ECM) protein. It is well known that the complex mixture of multiple components present in ECM can help both to maintain stemness or to promote differentiation of stromal cells following change in qualitative characteristics or concentrations. We investigated the possible correlation between ColXV expression and mineral matrix deposition by human mesenchymal stromal cells (hMSCs) with different osteogenic potential and by osteoblasts (hOBs) that are able to grow in culture medium with or without calcium. Analysing the osteogenic process, we have shown that ColXV basal levels are lower in cells less prone to osteo‐induction such as hMSCs from Wharton Jelly (hWJMSCs), compared to hMSCs that are prone to osteo‐induction such as those from the bone marrow (hBMMSCs). In the group of samples identified as ‘mineralized MSCs’, during successful osteogenic induction, ColXV protein continued to be detected at substantial levels until early stage of differentiation, but it significantly decreased and then disappeared at the end of culture when the matrix formed was completely calcified. The possibility to grow hOBs in culture medium without calcium corroborated the results obtained with hMSCs demonstrating that calcium deposits organized in a calcified matrix, and not calcium ‘*per se*’, negatively affected ColXV expression. As a whole, our data suggest that ColXV may participate in ECM organization in the early‐phases of the osteogenic process and that this is a prerequisite to promote the subsequent deposition of mineral matrix.

## Introduction

Mesenchymal stromal cells (MSCs) can differentiate into cells of mesodermal lineages such as cartilage, bone and adipose tissue 1. MSCs with different proliferation rate and differentiation potential can be isolated from various adult stem cell niches and extraembryonic tissues 2, 3, 4, 5. The donor‐related variability in MSCs differentiation potential has been well documented 3, 4, 5, 6, 7, 8, 9. This is a significant/problematic issue, because it can affect the interpretation of the data and limit the applicability and efficiency of cell‐based therapeutic approaches. Recently, a particular attention has been given to the identification of predictive markers for selecting MSCs with high differentiation potential and other desirable characteristics that would allow to obtain useful information for a therapeutic success 4, 10, 11, 12, 13. This is an important issue in the field of bone regenerative medicine concerning the production of tissue‐engineered constructs 14, 15, 16, 17, 18.

Osteogenic differentiation is a complex, tightly regulated process that is critical for proper bone formation during development and repair processes 19, 20. As MSCs pass through a temporal sequence of events towards differentiation, they lose their proliferative capacity, acquire the ability to respond to osteogenic stimuli and become committed to osteoblast lineage. They also support nascent osteoblast environment by ECM maturation and mineralization under a stringent control that is only partially understood 21. At the same time, MSCs must keep their ability to respond to the need of bone physiological remodelling. This condition is supported by ECM which is one of the major determinants of the structural integrity and functional properties of a stem cell niche, providing specific signals through different kind of molecular interactions with the cell surface. It is in fact widely understood that changes in ECM composition exert powerful control over many cellular phenomena, including stem cell differentiation and tissue remodelling 21, 22, 23. We are interested in studying still little investigated bone ECM components, in order to understand their possible participation to osteogenic differentiation and their potential role in sustaining bone repair. In this context, there is still much to understand about the role of certain collagens present in the matrix, their interactions with partner molecules or binding to specific receptors. Non‐fibrillar collagen (Col) XV is a chondroitin sulphate‐modified glycoprotein belonging to the multiplexin subfamily (multiple triple helix domains with interruptions) 24. Its expression is associated with vascular, neuronal, mesenchymal and some epithelial basement membrane (BM) in many tissues, indicating a probable function in the adhesion between BM and the underlying connective tissue stroma 24, 25. Its precise functions remain to be fully elucidated, even if evidence so far suggests that ColXV is involved in more sophisticated roles than just the molecular architecture of BM, particularly in the context of ECM organization and degradation 26, 27, 28.

By gene array profile and immunohistological analysis, we have previously identified ColXV as a novel human osteoblast (OB) matrix protein 29, and our interest is now to investigate a possible involvement of this type of collagen in triggering bone intracellular signalling pathways and regulating osteogenic cell growth and differentiation. In this study, we have investigated the impact of the presence of ColXV on mineral matrix deposition by hMSCs with different osteogenic potential and on human osteoblasts (hOBs) cells.

## Materials and methods

### Cell culture

hMSCs were isolated from two sources, human bone marrow (hBMMSC) and Wharton's jelly of umbilical cords (hWJMSC). hBMMSCs were obtained, as previously reported 30, from bone marrow aspirates harvested by the iliac crest, after obtaining the patients’ informed consent and the approval of the Istituto Ortopedico Rizzoli Ethics Committee. hWJMSCs were isolated from human umbilical cords collected after the mothers’ informed consent and the approval of the University of Ferrara and S. Anna Hospital Ethics Committee; samples were processed within 4 hrs, as already reported 31. Considering that osteogenic potential of hWJMSCs is usually related to the obstetric parameters 32, samples homogeneous for duration of the pregnancy (≥38 weeks) and birthweight (≥3.00 kg) were chosen. At subconfluence, cells were trypsinized and expanded or used immediately for *in vitro* experiments.

Flow cytometry analysis was performed on hBMMSC and hWJMSCs (at passage 1), fixed in 4% paraformaldehyde and incubated at 4°C for 30 min., with 5 μg/ml of the following monoclonal antibodies: anti‐human –CD34, –CD45 (DAKO Cytomation, Glostrup, Denmark), –CD31 (Chemicon International, Temecula, CA, USA), –CD73, –CD90, –CD146 (Becton Dickinson, Mountaine View, CA, USA), –CD105 (produced from the hybridoma cell line, clone SH2; ATCC, Rochville, MD, USA), –Runx2 (R&D System, Minneapolis, MN, USA), –alkaline phosphatase (ALP; Developmental Studies Hybridoma Bank, Iowa City, IA, USA), –osteocalcin (OC; R&D System),–bone sialoprotein (BSP, Immunodiagnostik, Bensheim, Germany), –collagen type 1 (Coll.1; Millipore, Temecula, CA, USA). Cells were washed twice and incubated with 2.4 μg/ml of a polyclonal rabbit antimouse (DAKO Cytomation) or goat anti‐rat (AbD Serotec, Oxford, UK) immunoglobulins/FITC conjugate antibody at 4°C for 30 min. After two final washes, cells were analysed using a FACStar plus Cytometer (Becton Dickinson). For isotype control, FITC‐coupled non‐specific mouse IgG was used instead of the primary antibody. Data were expressed as mean percentage of positive cells.

hWJMSC and hBMMSC were induced to osteogenic differentiation in DMEM high glucose (Euroclone S.p.A., Milan, Italy) supplemented with 10% foetal calf serum (FCS) (Euroclone S.p.A.), 100 mM ascorbic acid, 0.1 mM dexamethasone and 10 mM β‐glycerol phosphate (Sigma‐Aldrich, St. Louis, MO, USA). For the alizarin red staining (ARS), samples were fixed in ethanol 70%, stained with 40 mM, pH 4.2 Alizarin Red S solution (Sigma‐Aldrich), at room temperature for 10 min., rinsed in distilled water and washed in PBS on an orbital shaker at 40 r.p.m., to reduce non‐specific binding.

Human osteoblasts (hOBs) were isolated from trabecular bone chips and grown in DMEM/F12K (Gibco, Invitrogen Corporation, Indianapolis, IN, USA) without calcium supplementation with antibiotics, 25 μg/ml ascorbic acid, 4 mM glutamine (Sigma‐Aldrich) with or without 0.5, 1.3 and 2.6 mM extracellular CaCl_2_, as previously reported 33.

### Western blotting

For Western blot analysis, cells were washed twice with ice‐cold PBS and cell lysates were prepared as previously reported 31. For the processing of the media fractions, non‐induced or osteogenic‐induced cells (≥90% confluence) were starved in 0.1% FCS for 72 hrs before collection. Medium was clarified for 10 min. at 4700 × g and concentrated up to 50‐fold using Amicon Ultra‐15, 100 kD (Millipore, Billerica, MA, USA). The chondroitinase ABC digestions were performed for 90 min. at 37°C using 20 mU of enzyme as previously reported 34. Thirty microgram of each sample were electrophoresed on a 5–12% SDS‐polyacrylamide gel. Proteins were then transferred onto an Immobilon‐P PVDF membrane (Millipore, Billerica). After blocking with TBS‐0.1% Tween‐20 and 5% non‐fat dried milk, the membrane was probed with goat anti‐human collagen type 15 (ColXV) (1:200, clone C‐20; Santa Cruz, biotech, Dallas, TX, USA), rabbit anti‐human Runx2 antibody (1:1000, clone M‐70; Santa Cruz) washed and incubated with peroxidase‐conjugated anti‐goat or anti rabbit secondary antibody (Dako, Glostrup, Denmark) in 5% non‐fat dried milk. Immunocomplexes were detected using Immobilon Western Chemiluminescent HRP Substrate (Merck‐Millipore, Darmstadt, Germany). GAPDH, actin or IP3K were used to confirm equal protein loading. Densitometric analysis was performed by ImageJ software (NIH, USA, public domain available at: http://rsb.info.nih.gov/nih-image/).

### RNA extraction and qRT‐PCR

Total RNA was extracted using the RNeasy Plus Micro Kit (Qiagen, Hilden, Germany), according to the manufacturer's instructions. Briefly, cDNA was synthesized from total RNA (500 ng) in a 20 μl reaction volume using the TaqMan High Capacity cDNA Reverse Transcription Kit (Thermo Fisher Scientific, Waltham, MA, USA) as previously described 30.

Quantitative real‐time PCR was performed using gene expression master mix (Thermofisher) and analysed on CFX96 Real‐Time Detection System (Bio‐Rad laboratories, Hercules, CA, USA).

Assays‐On‐Demand kits (Thermo Fisher) for human COLXV and Runx2 were used. The expression level of cDNA samples was normalized to the expression of GAPDH, used as reference, with the formula 2^−ΔCt^. All reactions were performed in triplicate, and the experiments were repeated at least three times.

### Statistical analysis

The normal distribution of data was verified using the Kolmogorov–Smirnov test. In the case of single comparison, statistical significance was determined by unpaired Student's *t*‐test for normally distributed data and Mann–Whitney test for non‐normally distributed data. In the case of multiple comparisons, statistical significance was analysed by one‐way analysis of variance (anova) and Bonferroni *post hoc* test. Differences were considered statistically significant for *P*‐values ≤0.05.

## Results and Discussion

### ColXV and osteogenic potential of hMSCs

Fifty‐one samples of hBMMSCs and 65 samples of hWJMSCs were evaluated. Cells were characterized using conventional flow cytometric analysis with CD34, CD45, CD73, CD90, CD105, CD146 antibodies. Moreover, for each sample, the percentage of Runx2, ALP, OC, BSP and collagen type 1‐positive cells was also investigated. As reported in the Figure S1, all samples analysed showed a comparable phenotype except CD146, ALP and collagen type 1 which were expressed at significantly lower levels in the hWJMSCs compared to hBMMSCs.

We had previously found that osteogenically differentiated hBMMSCs and hWJMSCs showed a comparable increase in typical osteogenic markers such as Runx2, BSP, OC and collagen type 1 31, 35.

**Figure 1:**
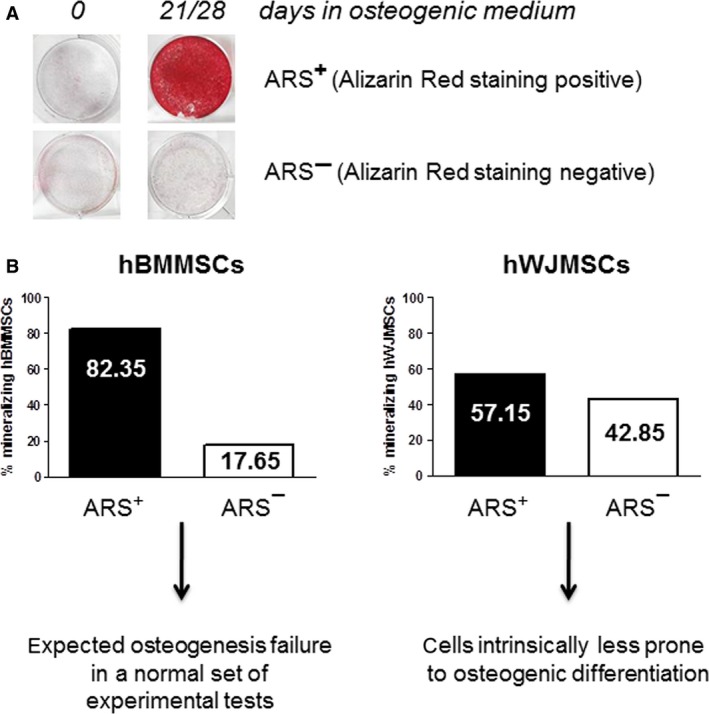
Secreted mineralized matrix by hMSCs. (**A**) hMSCs from bone marrow (hBMMSCs, *n* = 51) and from Wharton's jelly of umbilical cord (hWJMSCs, *n* = 65) were cultured for 21–28 days in osteogenic medium and analysed for their ability to secrete mineralized matrix by Alizarin Red staining (ARS). Samples were defined ARS^+^ when they reached a red peak staining at 21–28 days of culture, or ARS^−^ when they remained unstained. (**B**) Graphical representation of the percentage of mineralizing hBMMSCs and hWJMSCs samples that were ARS^+^ or ARS^−^.

However, when the functional *in vitro* end‐point reflecting advanced cell differentiation and osteogenic potential was assessed in terms of ECM mineralization, substantial differences between the various samples cultured in osteogenic medium were observed. Alizarin red staining (ARS) was used to evaluate the secreted mineralized matrix and showed differences among samples. We found samples creating a mineralizing microenvironment after 21–28 days, which we called ARS‐positive samples (ARS^+^) and samples that after 28 days were still not able to secrete mineralized matrix, which we called ARS‐negative samples (ARS^−^; Fig. 1A). These experiments demonstrated a higher percentage of ARS^+^ samples in hBMMSCs (82.35%) compared to hWJMSCs (57.15%; Fig. 1B).

**Figure 2:**
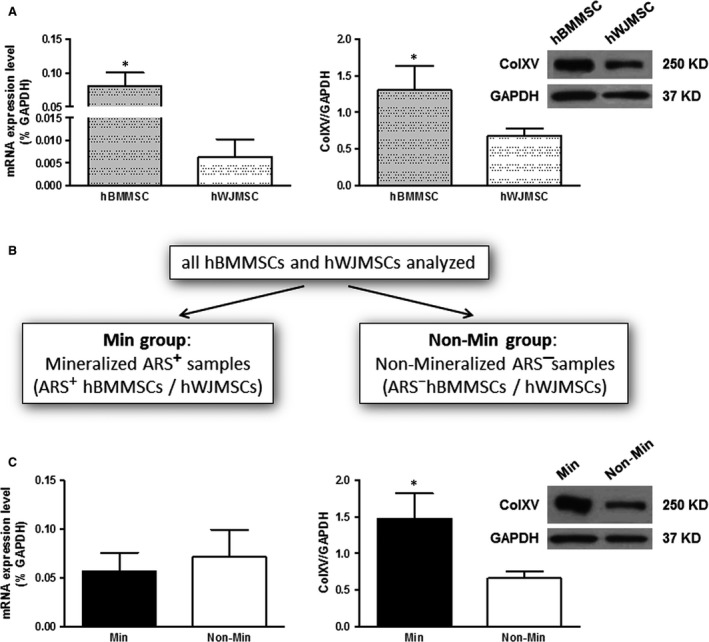
Analysis of ColXV basal levels performed by qRT‐PCR and Western blot. (**A**) ColXV evaluation in hBMMSCs and hWJMSCs at mRNA and protein level. mRNA data were expressed as % of the housekeeping gene GAPDH, and Western blot data were expressed as ColXV/GAPDH ratio. Representative Western blots are reported with densitometric analysis of all samples analysed. (**B, C**) ColXV expression was evaluated in the Min group (mineralized ARS^+^ hBMMSCs/hWJMSCs) and in the Non‐Min group (non‐mineralized ARS^−^ hBMMSCs/hWJMSCs). mRNA data were expressed as % of the housekeeping gene GAPDH, and Western blot data were expressed as ColXV/GAPDH ratio. Representative Western blots are reported with densitometric analysis of all samples analysed. Statistical analysis was performed comparing hBMMSCs *versus* hWJMSCs, or Min *versus* Non‐Min groups. *P ≤ 0.05 was considered statistically significant.

These observations are not a new concept as various evidence in literature highlighted interindividual and source‐dependent differences in the osteogenic potential of MSCs 11, 16, 36, 37. Therefore, it is reasonable to assume that hWJMSCs are intrinsically less prone to osteogenic differentiation compared to hBMMSCs for which the unsatisfactory results for mineralized ECM deposition may be ascribed to a normal variability in primary culture setting. To understand which elements and molecular mechanisms can contribute to the inability of the cells to develop mineralized matrix, it may be useful to identify new functional roles of specific molecules. It is known that when cells are unable to reach mineralization, ECM is disorganized, and this probably represents a restriction point for cell maturation. In this scenario, we focused on ColXV to understand whether it had a role in guiding the fate of MSCs and the mineral nodule formation. A first evidence for this hypothesis came from the analysis of ColXV basal levels which are significantly lower, both at mRNA and protein level, in cells less prone to osteo‐induction such as hWJMSCs, than in cells which are prone to osteo‐induction such as hBMMSCs (Fig. 2A). The expression of ColXV protein evaluated by Western blot of cell extracts corresponded to a band of the expected size (250‐kD) for the α1(XV) core protein 34.

To evaluate a possible correlation between the level of expression of ColXV and the ability of the cells to secrete mineralized matrix, we grouped together all hMSCs samples independently from their source (hBM or hWJ). Samples were then divided in two groups: mineralized ARS^+^ samples (Min group) and non‐mineralized ARS^−^ samples (Non‐Min group; Fig. 2B). We found that ColXV mRNA levels remained substantially unchanged between the two groups (Min *versus* Non‐Min group) while α1(XV) core protein level was significantly higher in the Min *versus* the Non‐Min group (Fig. 2C), highlighting that mRNA does not correlate with the same changes at protein level. This gave us the opportunity to make the following important observation. When we considered each case individually, we found an appreciable amount of the α1(XV) core protein also in samples with very low or scarcely measurable levels of mRNA (Fig. S2A and B, see samples *n =* 6, 7). Likewise, the sample with the higher level of mRNA (sample *n =* 4) is not the one with the highest protein content. This approach, based on the parallel evaluation of ColXV at mRNA and protein level, allows to reduce false‐positive or false‐negative samples and increases the possibility of finding a functional correlation between the expression of a putative marker, such as ColXV, and a specific osteo‐phenotype. Therefore, we confirmed that mRNA levels cannot be used as surrogates for corresponding protein levels without protein evaluation 38, 39. In particular, we showed that the more informative analysis for the expression of matrix proteins is once again represented by investigation of their protein level.

We also tested Runx2 expression as it has been shown that MSCs with higher basal level of mRNA for this factor have higher osteogenic differentiation ability 40. We found that both Min and Non‐Min samples did not show a correlation between basal level of Runx2 expression and osteogenic potential of the cells (Fig. S2B and C). Moreover, Runx2 expression did not follow the same trend of ColXV expression. This evidence confirms the importance of considering different parameters simultaneously to have a reliable prediction of the osteogenic potential 41.

Interestingly, we have not found any ColXV‐negative hMSC sample. Considering that ECM components including proteoglycans are often directly or indirectly involved in the regulation of the cell fate 42, our evidence supports the view that ColXV may contribute to the retention of features of a stromal cell phenotype by hMSCs.

### ColXV expression and mineral matrix deposition

**Figure 3:**
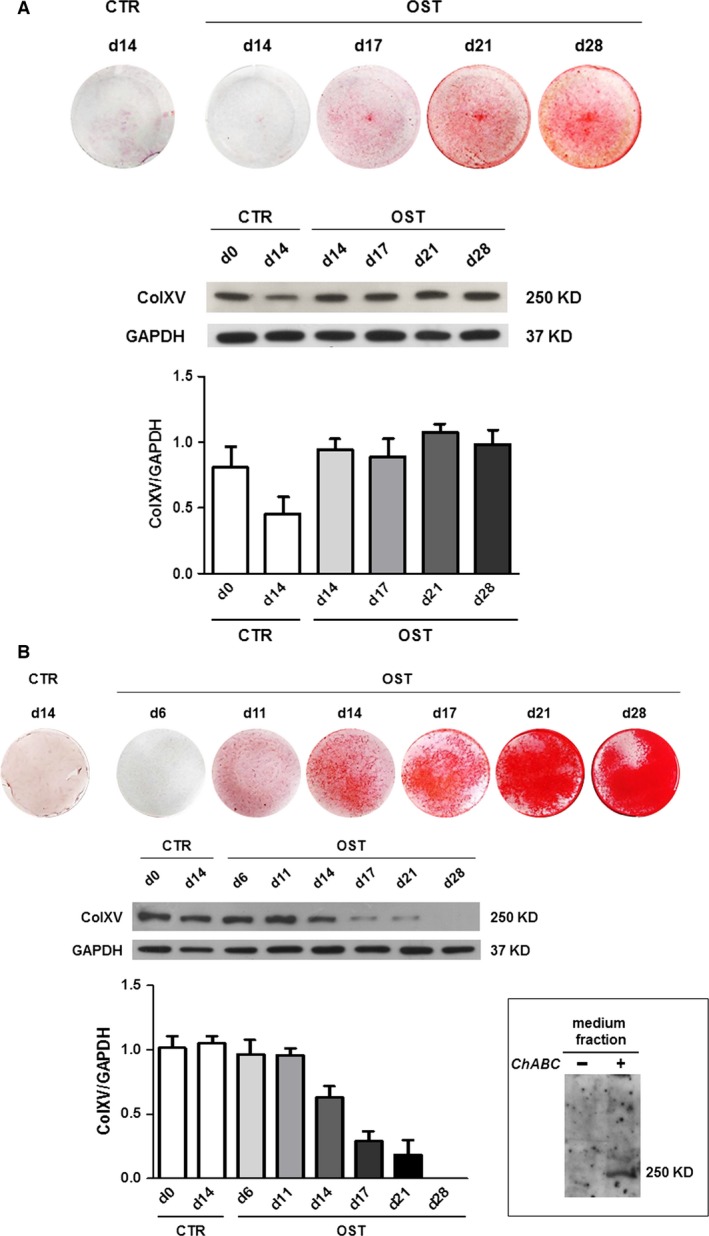
ColXV expression during osteogenic induction of hMSCs from Min group. The expression of ColXV was monitored at protein level during osteogenic induction (OST) at different time‐points until day 28 of culture and compared to the corresponding matrix mineralization status. The analysis of two Min representative samples with different degree of mineralization has been reported. The α1(XV) core protein expression was maintained stable in osteo‐differentiated hMSCs which did not reach a high degree of mineralization (**A**), while it significantly decreased when a strong mineralization was reached by a different sample (**B**). The densitometric analysis of each Western blot is reported, and data were expressed as ColXV/GAPDH ratio. CTR, control hMSCs from the same sample but not subjected to osteogenic induction. In the insert, the evaluation of ColXV expression in the medium fraction has been reported. Medium from hMSCs cultured for 72 hrs in the presence of 0.1% FCS was processed and electrophoresed on a 5% SDS‐polyacrylamide gel after incubation with (+) or without (−) chondroitinase ABC (ChABC).

We also monitored the expression of ColXV during the osteogenic induction of hMSCs to verify a possible relationship with the extent of deposition of mineralized matrix in hMSCs from the Min group. Considering the heterogeneity of hMSC cultures and donor variability, it is not surprising that even within the Min group, there is some difference in the degree of mineralization, as it is highlighted by a different ARS intensity. By comparing the level of mineralization by ARS and the expression of ColXV by Western blot on the same sample at different culture time‐points, we clearly demonstrated that the α1(XV) core protein was maintained at levels comparable to baseline, until the early stage of differentiation (Fig. 3A), and it significantly decreased and finally disappeared in samples reaching a high degree of mineralization (Fig. 3B). The baseline expression of ColXV has also been detected in the medium fraction from hMSCs cultured in presence of 0.1% FCS after chondroitinase digestion. This confirmed that ColXV is present as chondroitin sulphate proteoglycan functional molecule in ECM following secretion 34 (see Materials and methods and the insert in Fig. 3). We could not perform Western blot analysis of medium fraction during the 4 weeks of osteo‐differentiation as it precluded the use of serum‐low culture conditions. Therefore, to evaluate ColXV expression, we have to rely on core protein signal from cell extracts.

**Figure 4:**
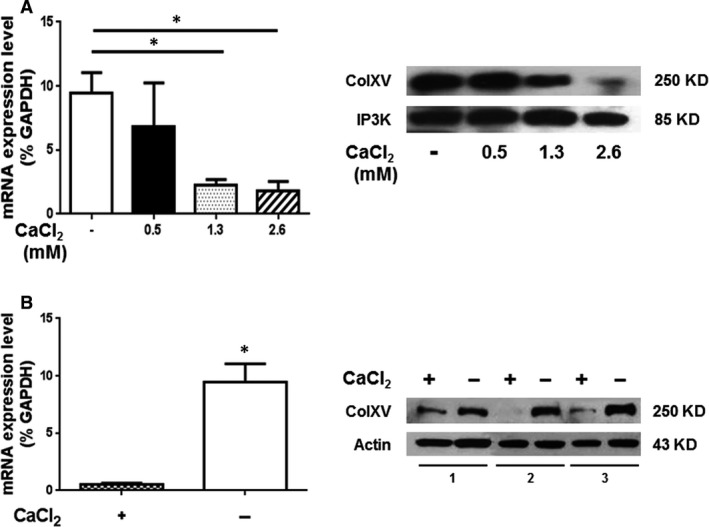
ColXV expression in osteoblasts. The analysis was performed by qRT‐PCR and Western blot in hOBs isolated and exposed to increasing concentrations of extracellular CaCl_2_ (0.5, 1.3 and 2.6 mM) for 48 hrs (**A**) or chronically maintained in calcium‐containing (1.3 mM) (+) or calcium‐free (−) medium for 7 days (**B**). Statistical analysis was performed in calcium‐free *versus* calcium‐containing medium condition. *P ≤ 0.05 was considered statistically significant.

This evidence suggests that ColXV might act as an ECM organizer in the early‐phases of the osteogenic process and that this should be a prerequisite to promote the subsequent deposition of mineral matrix. At the end of *in vitro* osteogenic differentiation when the microenvironment is completely calcified, α1(XV) core protein disappeared. These data, together with our previous immunohistological analysis 29, support the hypothesis that ColXV expression must be downmodulated in the presence of high amounts of extracellular calcium deposits in the mineralized matrix. In fact, as we previously demonstrated in bone tissue biopsies, ColXV was positive on osteoblasts lining bone trabeculae and negative on osteocytes 29. Moreover, this hypothesis is also supported by our previous data on hOBs isolated directly from bone chips. These cells, when chronically stimulated with different calcium concentrations, showed a significant increase in osteocalcin‐osteogenic marker and ECM mineralization, whereas ColXV mRNA was downmodulated 33. We also confirmed these data at protein level by Western blot analysis (Fig. 4A) on hOBs after exposure to increasing concentrations of extracellular CaCl_2_ (0.5, 1.3 and 2.6 mM). Moreover, we took advantage of the ability of hOBs to grow in culture medium with or without calcium to better understand the influence of calcium on ColXV. As shown in Figure 4B, we found that hOBs cultured in medium without calcium expressed high amounts of ColXV both at mRNA and protein level compared to hOBs obtained in the same manner but grown in conventional culture medium containing calcium.

By contrast, hMSCs showed a different scenario. The inability of hMSCs to proliferate and differentiate in culture medium without calcium has been in fact established 43. This indicates that the presence of calcium is ‘*per se*’ essential to allow hMSCs to grow and move towards the osteogenic lineage, but it has no influence on the expression of ColXV and the osteogenic potential of hMSCs. In fact, we demonstrated that hMSCs grown in the same culture medium have different ColXV levels and exhibit different performance in terms of ability to deposit mineral matrix (Fig. 2 and Fig. S2). Conversely, when calcium deposits in the extracellular calcified matrix were abundantly produced by mature cells (terminally osteo‐differentiated MSCs, Fig. 3B, or OBs 33, Fig. 4) we observed a substantial decrease in α1(XV) core protein expression.

**Figure 5:**
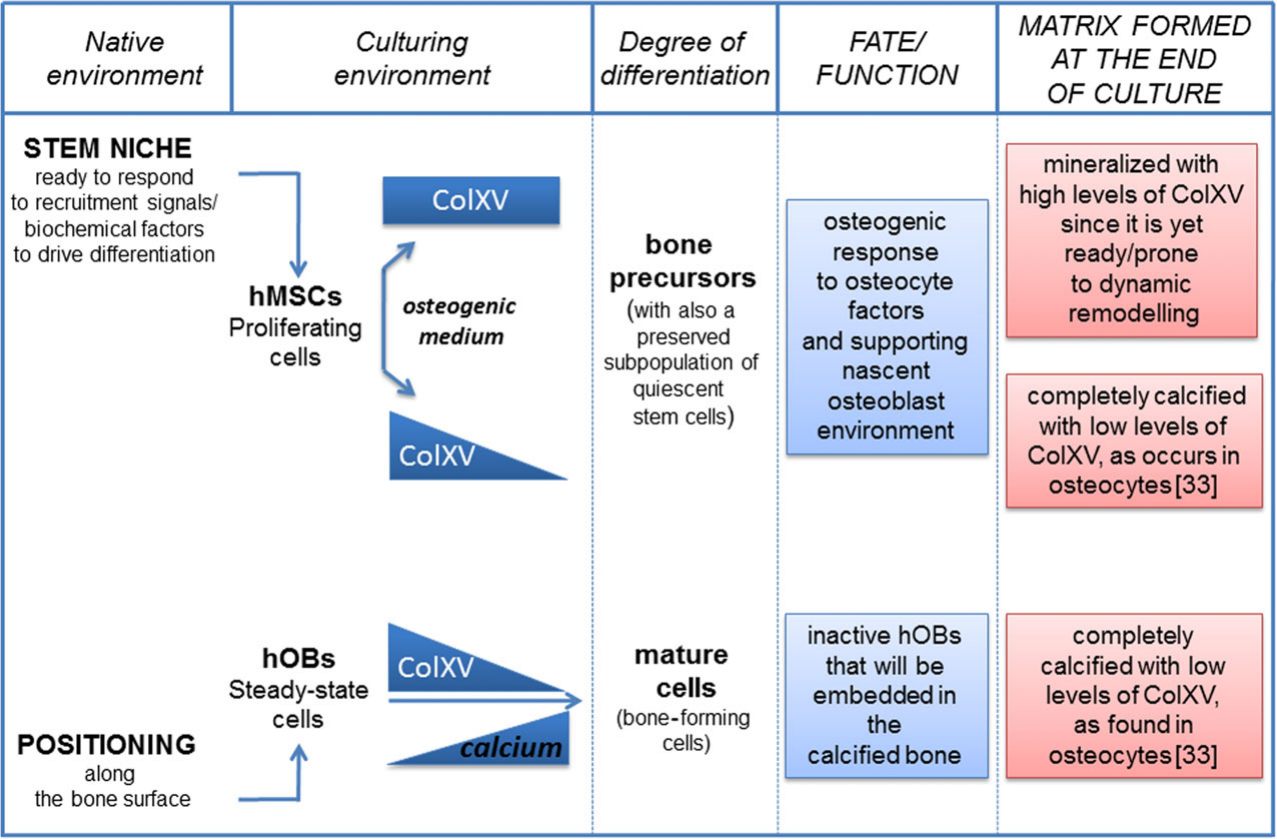
Schematic representation of a possible correlation between ColXV expression levels and mineral matrix deposition in hMSCs with different status of maturation and in hOBs that are able to grow in culture medium with or without calcium. Nearly constant expression of ColXV in those hMSCs which osteo‐differentiated but did not produce a completely calcified matrix could be useful to keep cells in a prone and responsive state to osteogenic differentiation stimuli, allowing bone remodelling or regeneration. Conversely, cells at the end of differentiation, such as osteoblasts or completely osteo‐differentiated hMSCs, are present in a completely calcified extracellular matrix with low or undetectable levels of ColXV.

In Figure 5, we summarized in a cartoon our hypothesis on the degree of mineralization associated to ColXV expression, evidencing a peculiar fate of MSCs and OBs in favouring dynamic bone remodelling or in maintaining mature cell in calcified bone areas. In particular, we believe that the nearly constant levels of ColXV during the osteogenic process could be useful to keep the hMSCs prone to dynamic remodelling and capable of responding to those signals supporting nascent osteoblast environment or repair of a damage. It is in fact well established that the complex mixture of multiple components present in ECM helps maintaining MSCs stemness in the MSC niche or to promote differentiation following changes of qualitative characteristics or concentrations 21, 44, 45, 46.

On the basis of our preliminary data, it will be interesting to understand how ColXV interacts with the extracellular environment, in particular, whether ColXV is a key accessory protein involved in cell– ECM interactions, or a crucial component directly involved in MSCs or OBs behaviour. Moreover, it will be interesting to explore how ColXV can affect intracellular signalling in response to differentiation signals, injury, physiological bone remodelling and development, and proper mineralization process initiated as an intracellular event.

It is known that collagens and their bioactive fragments (released by proteolytic cleavage) play critical functional roles in many physiological and pathological processes such as development, angiogenesis, tissue repair, tumour growth and metastasis 47, supporting the hypothesis that ColXV is a multifunctional collagen–proteoglycan with characteristics different from what originally believed. Therefore, further studies are needed to understand which signals control the expression of ColXV and the production of its proteolytically processed C‐terminal fragment that functions as an endostatin by inhibiting endothelial cell migration and angiogenesis 48.

## Conflict of interest

The authors confirm that there are no conflict of interests.

## Supporting information


**Figure S1.** hBMMSCs and hWJMSCs were characterized by flow cytometry for the expression of mesenchymal markers (CD73, CD90, CD105, CD146), hematopoietic markers (CD34, CD45) and typical osteogenic markers (Runx2, ALP, OC, BSP and Coll.1).

**Figure S2.** Analysis of ColXV and Runx2 basal levels performed by qRT‐PCR (A, C) and Western blot (B) on individual representative cases (four Min and four Non‐Min). mRNA data were expressed as % of the housekeeping gene GAPDH, Western blot data were expressed as ColXV/GAPDH ratio.
