# Supplementary figures and images for: Collagen type XV and the ‘osteogenic status’
Source: J Cell Mol Med. 2017 Mar 22;21(9):2236–44. doi: 10.1111/jcmm.13137 (PMC5571525; doi:10.1111/jcmm.13137)

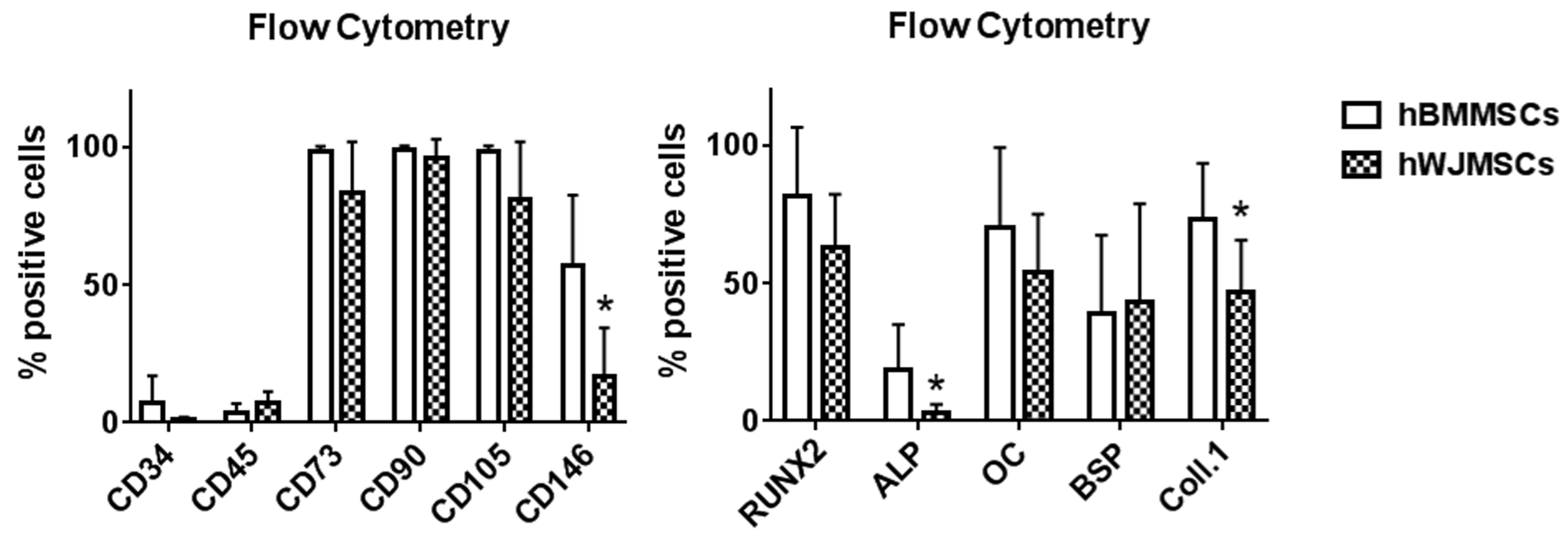

Supplement: Supplementary file 1 — Figure S1. hBMMSCs and hWJMSCs were characterized by flow cytometry for the expression of mesenchymal markers (CD73, CD90, CD105, CD146), hematopoietic markers (CD34, CD45) and typical osteogenic markers (Runx2, ALP, OC, BSP and Coll.1). [file JCMM-21-2236-s001.tiff]

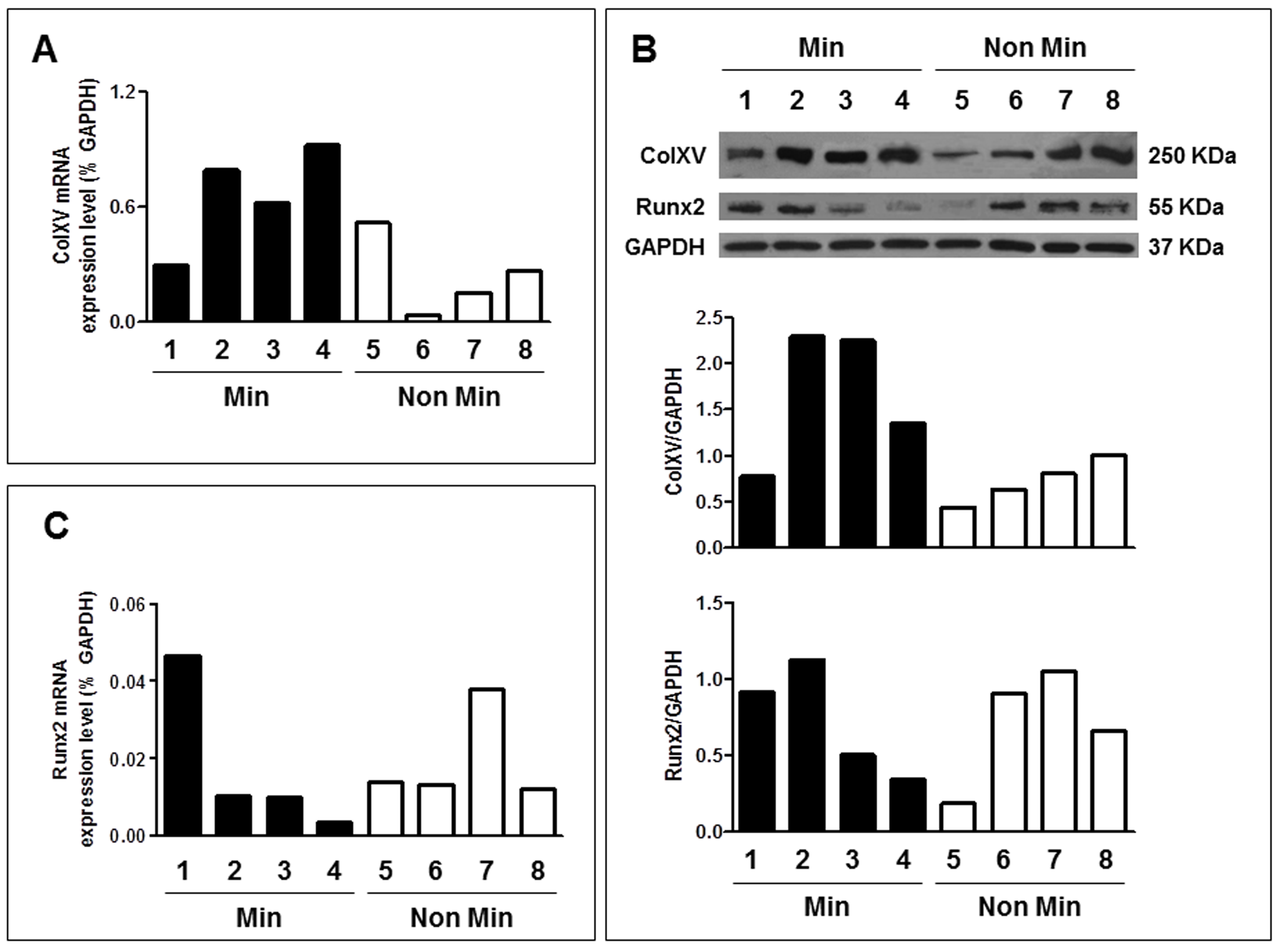

Supplement: Supplementary file 2 — Figure S2. Analysis of ColXV and Runx2 basal levels performed by qRT‐PCR (A, C) and Western blot (B) on individual representative cases (four Min and four Non‐Min). mRNA data were expressed as % of the housekeeping gene GAPDH, Western blot data were expressed as ColXV/GAPDH ratio. [file JCMM-21-2236-s002.tiff]
